# Acute ingestion of Ibuprofen does not influence the release of IL-6 or improve self-paced exercise in the heat despite altering cortical activity

**DOI:** 10.1007/s00421-024-05452-z

**Published:** 2024-03-06

**Authors:** Nicole T. Vargas, Caroline V. Robertson, Frank E. Marino

**Affiliations:** 1 School of Medicine and Psychology, College of Health and Medicine, Australian National University, 54 Mills Rd, Florey Building, Canberra, ACT 2601 Australia; 2 Griffith Centre For Mental Health and ALIVE National Centre for Mental Health Research Translation, Griffith University, Nathan, QLD Australia; 3 School of Rural Medicine and Research Group for Human Adaptation, Exercise and Health, Charles Sturt University, Orange, NSW 2890 Australia

**Keywords:** Inflammation, Electroencephalography, Cycling, Physical activity, Fatigue

## Abstract

The present study tested the hypothesis that ingesting 800 mg Ibuprofen prior to self-paced cycling at a fixed rating of perceived exertion (RPE) improves performance by attenuating the release of Interleukin (IL)-6 and its signalling molecules, whilst simultaneously modulating cortical activity and cerebral oxygenation to the brain. Eight healthy, recreationally active males ingested 800 mg Ibuprofen or a placebo ~ 1 h prior to performing fixed RPE cycling for 60 min in 35 °C and 60% relative humidity at an intensity of hard to very hard (RPE = 16) with intermittent maximal (RPE = 20) sprints every 10 min. Power output (PO), core and mean skin temperatures (*T*_c_, *T*_sk_), respectively, and heart rate (HR) were measured continuously. Electroencephalography (EEG) recordings at the frontal (Fz), motor (Cz) and Parietal (Pz) areas (90 s) were collected every 5 min. IL-6, soluble glycoprotein receptor (sgp130) and IL-6 receptor (R) were collected at pre-, 30 min and immediately post-exercise. Mean PO, HR, *T*_c_ and *T*_sk_, and RPE were not different between trials (*P* ≥ 0.33). At end-exercise, the change in IL-6, sgp130 and sIL-6R was not different between trials (*P* ≥ 0.12). The increase in *α* and *β* activity did not differ in any cortices between trials (*P* ≥ 0.07); however, there was a significant reduction in *α*/*β* activity in the Ibuprofen compared to placebo trials at all sites (*P* ≤ 0.05). Ingesting a maximal, over-the-counter dose of Ibuprofen prior to exercise in the heat does not attenuate the release of IL-6, nor improve performance, but may influence cortical activity evidenced by a greater reduction in *α*/*β* activity.

## Introduction

Exercise in the heat has been shown to elevate the circulating cytokine interleukin-6 (IL-6) compared with exercise in thermoneutral conditions, likely via tissues other than mononuclear cells (e.g. skeletal muscles or other lipopolysaccharide induced causes) (Rhind et al. 2009; Starkie et al. 2005). It is also suggested that an elevated cytokine response during exertional heat stress may augment increases in core temperature (*T*_c_) independent of metabolic heat production, increase signalling to the central nervous system (CNS) and result in behavioural modifications (Rhind et al. 2009; Lim and Mackinnon 2006). For example, centrally injecting IL-6 and IL-1β into conscious rats not only increases *T*_c_, but also decreases wheel running (Harden et al. 2014). Moreover, it has also been postulated that IL-6 can signal the CNS through its soluble (s) glycoprotein (gp)-130 and membrane-bound receptor (IL-6R) (Gray et al. 2009; Jones and Rose-John 2002). As such, it has been proposed that IL-6 may be an important molecule for communication between the periphery and the CNS during exertional heat stress (Vargas and Marino 2016).

Ingesting acute doses of analgesic drugs (e.g. Ibuprofen or acetaminophen) to counter the inflammatory response prior to, or after exercise training and competition, is a common practise amongst recreational and elite athletes (Brewer et al. 2014; Gorski et al. 2011). Ibuprofen ingestion prior to exercise has been shown to attenuate increases in IL-6 after moderate, mixed-mode intensity exercise (Wherry et al. 2021), yet increases in cytokine production have been noted following Ibuprofen ingestion during an ultramarathon race (Nieman et al. 2005). Although specific mechanisms remain unknown, the contrasting outcomes are likely to due to the differing modalities and exercise durations used within the aforementioned studies. In rats, Ibuprofen has been identified as an ergogenic aid by increasing exercise time to exhaustion (Lima et al. 2015). The exact mechanism of action by which Ibuprofen may act as an ergogenic aid remains unknown, but it has been suggested that there is protection against the overproduction of pro-inflammatory cytokines, which in turn may alter the neuronal functions related to exercise-induced fatigue (Lima et al. 2015). Although speculative, changes in the inflammatory cascade due to Ibuprofen may result in the inhibition of trans-signalling of IL-6 to neuronal areas through sgp130, which blocks the sIL-6R/IL-6 complex from signalling through a membrane-bound gp130 (Rose-John 2012). It follows that Ibuprofen may attenuate the fatiguing effects of endurance exercise and could improve exercise performance during heat stress.

Changes in cortical activity measured through electroencephalography (EEG) occur with exercise and due to ingestion of Ibuprofen. Increased alpha (*α*) wave activity is preferentially maintained in the prefrontal cortex during a maximal graded exercise test to exhaustion (Robertson and Marino 2015), and is associated with reductions in power output during self-paced exercise (Vargas and Marino 2018). It has also been shown that beta (*β*) wave activity is reduced during exercise in the heat, and the ratio of *α*/*β* wave activity increases during exercise hyperthermia (Nielsen et al. 2001) compared to a longer but less thermally demanding exposure (Ftaiti et al. 2010), although this was not evident in our previous study (Vargas and Marino 2018). Nevertheless, Ibuprofen ingestion also results in inhibition of EEG signals in rats (Wallenstein 1985) and has been shown to alter delta (*δ*) waves of Alzheimer’s patients after chronic use (Babiloni et al. 2009).

In addition to cortical activity, cerebral haemodynamics have previously been identified as a marker of neuronal activity (Ogoh and Ainslie 2009; Perrey 2008). Total haemoglobin content (HbTot) has been shown to increase and remain stable during a 5 km running time trial until the final 0.5 km, where deoxyhaemoglobin (HHb) increases in conjunction with increased effort, thus leading to reductions in the overall haemoglobin difference (HbDiff) (Billaut et al. 2010). It has also been shown that heat stress reduces cerebral oxygenation during fixed intensity, submaximal cycling (Rasmussen et al. 2010). To our knowledge, the combined effects of Ibuprofen and exercise on cerebral oxygenation and cortical activity have not yet been studied. However, given that Ibuprofen may concomitantly exert its effects centrally, it is possible that information processing and overall perception of effort may be altered through sensory feedback.

The aims of this study were to examine the effect of an acute, maximal, over-the-counter dose of Ibuprofen prior to a bout of self-paced exercise in the heat on performance (i.e. power output), the release of IL-6 and its soluble receptors, and changes in cortical activity and cerebral oxygenation during a self-paced exercise protocol in the heat. We hypothesised that ingestion of Ibuprofen would result in greater power output, attenuated IL-6 and sIL-6R, but increased sgp130 responses. A secondary hypothesis was that ingestion of Ibuprofen prior to exercise would result in attenuated *α* and increased *β* wave activity (*α*/*β*), and augmented cerebral oxygenation compared to a placebo.

## Methods

### Ethics approval

The study was approved by the University Research and Human Ethics Committee. This study conformed to standards set by the latest revision of the Declaration of Helsinki, except that the study was not a clinical trial and thus was not registered as such. All participants provided verbal and written consent prior to participating in the study.

### Participants

An a priori power calculation was not completed prior to commencing this study as to the best of our knowledge, there are no prior studies which report the effects of Ibuprofen ingestion on our primary outcome (power output) during a short (~ 60 min) exercise protocol in humans. Thus, we aimed to collect a total of 15 participants; however, due to timing and funding constraints, we completed the study with a sample size of 8.

Eight healthy, recreationally active (Pauw et al. 2013) males (24 ± 6 years, 183 ± 9 cm, 81.2 ± 12.5 kg, 44.0 ± 5.1 mL/kg·min^−1^) participated in this study and were screened for current and previous exercise and disease history using the adult pre-screening exercise tool (APSS) (Exercise and Sports Science Australia; ESSA). Exclusion criteria included any previous or current conditions, injury or medications likely to alter the neurophysiological state of the brain. Further exclusion criteria were any reoccurring or a recent (< 3 weeks) bout of influenza illness, taking anti-inflammatory or any other medications known to interfere with a normal inflammatory response or individuals with rheumatoid arthritis, recent and/or current periodontal disease, and any other conditions associated with an altered inflammatory state.

### Study design

Participants reported to the laboratory on three occasions, consisting of one familiarisation and two experimental trials performed in a crossover design separated by at least 7–10 days. A maximal aerobic test (*V*O_2peak_) was performed during the familiarisation visit, followed by familiarisation of the exercise protocol and equipment used in the subsequent trials. During the experimental trials, subjects were required to cycle at a clamped RPE of 16 (i.e. ‘very hard’), according to the 6–20 Borg RPE scale (6 = no exertion, 20 = maximal exertion) (4) in a climate chamber set at 35 °C, 60% relative humidity.

The clamped RPE methodology was used because it has been shown that at a fixed RPE, self-selected power output is reduced according to the prevailing environmental conditions (Tucker 2009), and that α wave activity is associated with reductions in power output (Vargas and Marino 2018). In addition, thermoregulatory responses and self-selected exercise intensity are modulated by RPE (Schlader et al. 2011a). Therefore, clamping the RPE provided a predictable outcome for these variables (Schlader et al. 2011a, 2011b). A modified version of the clamped RPE protocol, extended to 60 min of exercise, was employed in the present study to elicit predictable reductions in power output as in our prior work (Vargas and Marino 2018). In this protocol, participants were required to perform a maximal 30 s sprint at the end of each 10 min period to increase exercise intensity for the purpose of obtaining an adequate intensity for cytokine release. Pilot testing of this protocol revealed an intra-individual and inter-individual CV of 10% and 3%, respectively, for power output, and 2% and 3%, respectively, for heart rate. Although the CV for power output was slightly higher than deemed acceptable, that for the physiological outcomes was in an acceptable range. Mean power output of the steady-state bouts of exercise and of each 30 s sprint were used as indicators of exertional fatigue.

### Familiarisation trial

Subjects reported to the laboratory for baseline measures of body mass and stature and a VO_2peak_ test to determine fitness level and peak power output (PPO) on a cycle ergometer (Veletron DynaFit Pro, RacerMate Inc., WA, USA). All ergometer measurements were recorded for future use to decrease within-subject variability on the equipment. During the VO_2peak_ test, subjects completed a 5 min warm up at light–moderate intensity followed by 2 min of rest. The test commenced at 100 W and increased by 20 W every minute until volitional exhaustion, defined by the inability to maintain a cadence above 60, or the subject voluntarily terminating the test.

Following a sufficient rest period and explanation of the remaining testing procedures (~ 30 min), subjects cycled on an identical ergometer in a heat chamber for an initial 10 min at variable RPE ratings to ensure understanding of both how to change the resistance on the ergometer and how to interpret the RPE scale. After familiarisation of different RPE ratings, subjects were asked to cycle at an RPE of 16 (hard-very hard) (Borg 1982) for the remaining 20 min. Subjects also performed a maximal (RPE = 20) sprint every 10 min. Following 30 min of familiarisation with the protocol, subjects cycled at a comfortable pace to cool down for 5 min and were instructed that a reduction in power during the test was okay, provided that they were maintaining the required RPE throughout the whole protocol.

### Experimental trial

Subjects arrived at the laboratory 2 h after eating a small meal limited in carbohydrates. Due to the potential interaction between carbohydrates and the release of IL-6, participants were restricted to eating the following items: eggs, ham, bacon, sausage, protein shakes with carbohydrate concentration < 1%, fish, cheese, organic or no sugar added peanut butter limited to 2 tbsp, tofu, or hummus limited to 2 tbsp. Participants also arrived having avoided physical activity for at least 36 h and caffeine and alcohol at least 24 h prior. A pre-trial food and activity log was maintained by each participant for 48 h prior to the first experimental trial and was followed for the 48 h up to the subsequent trial which was also confirmed upon arrival. Immediately upon arriving at the laboratory, subjects who ingested either 800 mg of Ibuprofen, or a placebo (gluten-free flour) tablet with water to ensure peak half-life (1.8–2.0 h) concentrations, were reached approximately halfway through the exercise protocol (Bushra and Aslam 2010). Following instrumentation (~ 40 min), subjects entered the climate chamber and sat for 5 min for a resting baseline measure prior to commencing the 60 min cycling protocol.

### Data collection

Baseline measures for each experimental session consisted of resting heart rate (HR), *T*_c_ and skin temperature (*T*_sk_), a 15 mL blood sample for IL-6, sIL-6R and sgp-130, neuronal indices (EEG and cerebral oxygenation via near-infrared spectroscopy (NIRS) at the frontal cortex) and RPE. A 15 mL blood sample was collected halfway through the protocol at 30 min, immediately following the cessation of exercise at 60 min and after 1 h passive recovery. A 90 s EEG recording and RPE were collected every 5 min during exercise. Power output (PO), HR, *T*_sk_ and cerebral oxygenation were recorded continuously. A post-exercise EEG measure was taken immediately after the exercise protocol ended.

Power output (PO; RacerMate, Seattle, WA) and HR (Polar, Kempele, Finland) were measured continuously at a sampling rate of 2000 Hz. HR data were also manually recorded every 5 min using an accompanying sports watch (RS300X, Polar, Kempele, Finland) for accuracy. Mean PO during steady-state periods was calculated as the average total PO for the steady-state time (e.g. min 0–9:30 every 10 min) during the 60 min cycling protocol. Mean PO during the sprint was calculated during the 30 s sprint at the end of every 10 min period (e.g. min 9:30–10:00).

Approximately 6 h prior to reporting to the laboratory, subjects ingested a wireless telemetry pill (Jonah™ Core body temperature capsule, VitalSense Company, Inc., Bend, OR) for the measure of *T*_c_. After ingesting the pill, subjects were only permitted their small breakfast meal, and water prior to the trial. Upon arrival to the laboratory, a small amount of water was consumed with the Ibuprofen or placebo tablet. Participants were able to ingest water ad libitum throughout the trial. Measures of *T*_sk_ were collected using Four Thermodata TDHC thermologgers (Brisbane, QLD) fixed to the skin (Opsite Flexifix, Smith & Nephew, AU) on the left side at the bicep, chest, mid-thigh and mid-calf area with data were sampled every minute. Mean *T*_sk_ was calculated as 0.2 × (Thigh + Calf) + 0.3 ×  (Bicep + Chest) (Ranamathan 1976).

To determine plasma concentration of IL-6, sIL-6R and sgp-130, 15 mL blood was collected at each time point using a standard 22 g cannula (Becton Dickinson, NJ, USA) that remained in the arm for the duration of the protocol. Approximately 2 mL of the sample was discarded and 13 mL was transferred immediately to an ethylenediaminetetraacetic acid (EDTA) tube and centrifuged at 4 °C, 3500 rpm^−1^. After centrifuging for 15 min, 1 mL of plasma was immediately aliquoted into two microfuge tubes and stored at − 80 °C. Blood samples were analysed in duplicate using Merck Millipore Multiplex Assay human cytokine/chemokine magnetic bead panel kit for IL-6 (intra-assay coefficient of variation (CV): 12%; inter-assay CV: 11%, HCYTOMAG-60 K, Magpix, Luminex, Austin TX) sgp130 (intra-assay CV: 16%; inter-assay CV: 7%) and sIL-6R (intra-assay CV: 7%; inter-assay CV: 7%, HSCRMAG-32 K, Magpix, Luminex, Austin TX).

Subjects were fitted with a 20-channel wireless EEG system (BAlertX24, ABM, CA) based on the circumference of their head at a level just superior to the glabella, the measure of the distance from the glabella to the occipital protuberance and between external acoustic meatus. EEG electrodes were filled with Synapse Conductive Electrode Cream (ABM, CA). All electrodes and the paired mastoid reference sites were cleaned and abraded prior to checking scalp-electrode impedance (Ω). The impedance of each EEG site of interest was maintained below 20 kΩ as directed by the manufacturer. Signals were collected at a sample rate of 256 Hz and acquired wirelessly across an RF link via RS232 interface. Data were sampled with a bandpass filter from 0.5 to 65 Hz and raw signals monitored during rest and exercise. EEG signals were collected from all 20-electrode sites but only those from the frontal cortex (FC; F3, F4, Fz), motor cortex (MC; C3, C4, CZ) and parietal cortex (PC; P3, P4, Pz) were used for analysis. Data were processed and analysed using B-Alert Lab (Version 2.0, ABM, CA). Eye blink and muscle artefact was removed by B-Alert decontamination algorithms and each 90 s recording was manually inspected for artefact (Polyman, version 1.153.1065). Decontaminated data were then fast Fourier transformed with a Kaiser window applied to give mean power spectral density (PSD) for *α* (8–12 Hz) and *β* (13–30 Hz) frequencies. The percent change from baseline PSD in each measured site for both *α* and *β* frequencies was averaged for each 90 s snapshot.

Measures of oxy- (O_2_Hb) and deoxy- (Hhb) haemoglobin from the left prefrontal cortex (PFC) were collected from two optodes placed 40 mm apart using double side adhesive tape for the measure of cerebral blood flow using near-infrared spectroscopy (NIRS) (Niro-200 × Hamamatsu Photonics, Hamamatsu, Japan). Data were obtained at a frequency of 60 Hz using wavelengths of 735, 810 and 850 nm to calculate the change (Δ) in O_2_Hb and ΔHhb. Total haemoglobin concentration (Hb_total_) was calculated as the sum of O_2_Hb and Hhb. The haemoglobin difference (Hb_difference_) was calculated by subtracting Hhb from O_2_Hb. These indices are reliable indicators of cerebral blood flow and tissue deoxygenation, respectively (3, 33).

### Data and statistical analysis

Continually recorded data (i.e. PO, T_c_, HR, T_sk_) were binned as 60 s averages every 5 min to represent steady-state exercise at 5, 15, 25, 35, 45 and 55 min. Mean PO during the sprint was calculated over the full 30 s duration for each sprint. EEG and NIRS data were analysed only during the steady-state exercise bouts and averaged over the full 90 s snapshot. All data are reported as absolute data except EEG (reported as a percent change from baseline to account for daily variation within an individual) and NIRS (reported as absolute change from baseline). These data were analysed using a two-way (condition x time) repeated measures or mixed model (where data were missing) ANOVA. All data were checked for normality using a Shapiro–Wilk test. Most data sets (59 out of 64) met normality. Due to the small sample size, whether the data meet a normal Gaussian distribution is unlikely, therefore we continued with parametric testing. Violations of sphericity were accounted for using the Geisser–Greenhouse correction. When an ANOVA revealed a significant *F* test for main effect or interaction, Sidak post hoc analyses were performed. One-tailed paired *t *tests were used to determine differences in total work, average RPE, mean sprint PO, and end-trial change in *T*_c_, *T*_sk_, IL-6, sgp130 and sIL-6R. A one-tailed paired *t *test was also used to determine differences in average trial EEG and NIRS activity between conditions. All analyses were performed using GraphPad Prism Software (V6, La Jolla, CA). Statistical significance was set at *P* ≤ 0.05. Cohen’s *d* effect sizes were calculated and reported for the primary outcomes with small effect = 0.2; medim effect = 0.5; large effect ≥ 0.8.

## Results

### Performance, cardiovascular, thermal and perceptual responses

**Fig. 1:**
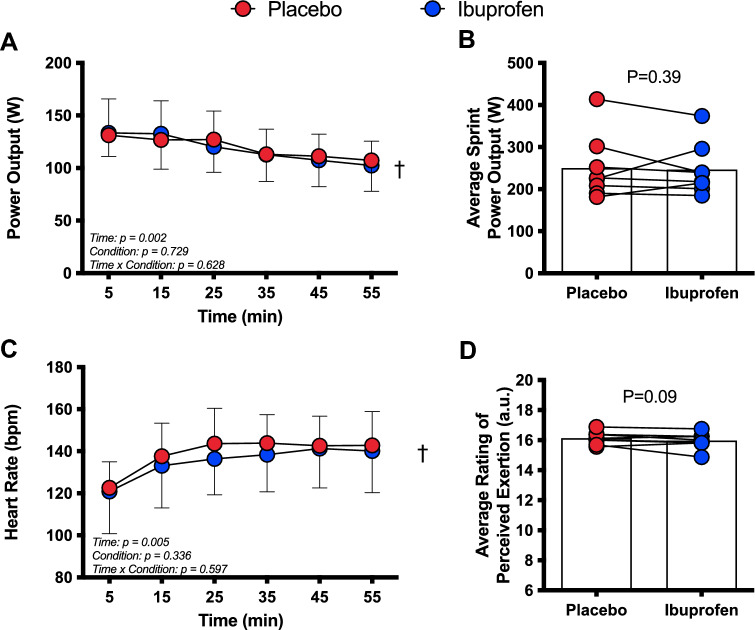
Mean power output (**A**), average sprint power output (**B**), meat heart rate (**C**) and average rating of perceived exertion (**D**) presented as *n* = 8, mean ± SD during 60 min cycling in the heat after ingestion of either a placebo (red) or Ibuprofen (blue). ^†^Main effect of time (*P* < 0.005)

**Fig. 2:**
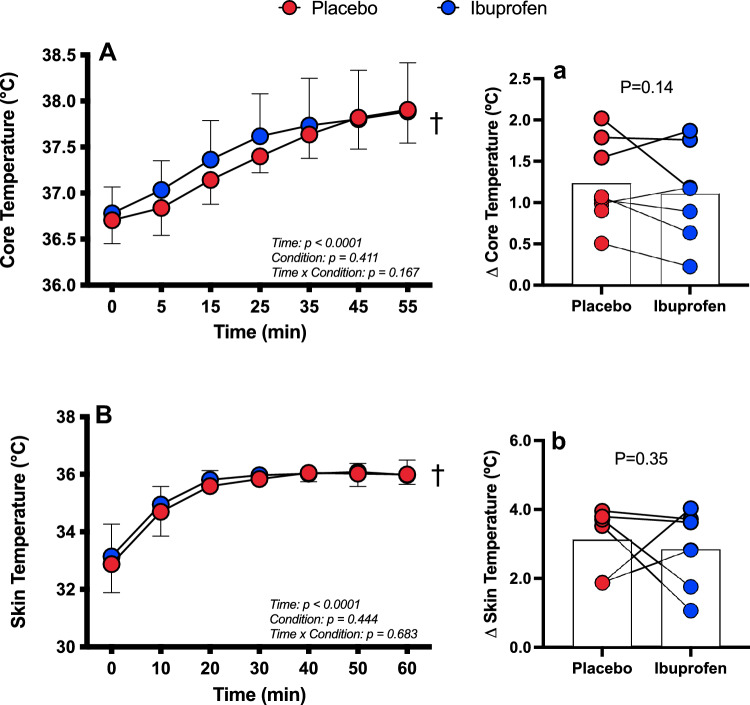
Average core (**A**) and delta core temperature (**a**) and average mean skin (**B**) and delta mean skin temperature (**b**). *N* = 8 for core temperature data and *n* = 6 for skin temperature data due to equipment error. Data presented as mean ± SD during 60 min cycling in the heat after ingestion of either placebo (red) or Ibuprofen (blue). ^†^Main effect for time (*P* < 0.0001)

The absolute change in PO from the first 5 min was not different between Ibuprofen and placebo (*P* ≥ 0.72, Fig. 1A). Likewise, there was no interaction or main effect for condition for absolute and average PO (*P* ≥ 0.63, ES = 0.12), but there was a main effect for time whereby average PO decreased throughout the trials (*P* < 0.003). Total work was not different between trials (IBU 394.9 ± 93.43; PLA 388.4 ± 93.43, *P* = 0.33, ES = 0.07). There was no interaction (*P* = 0.76), main effect for condition (*P* = 0.79) or time (*P* = 0.78) for mean sprint PO at the end of each 10 min period (Fig. 1B). There was only a main effect of time for HR, increasing throughout (*P* < 0.005, Fig. 1C). There was also no interaction (*P* = 0.41) or main effect of condition (*P* = 0.39) for RPE; however, it increased in both trials over time (*P* < 0.0001, Fig. 1D). Accordingly, there were no significant differences between average trial RPE (*P* = 0.09, ES = 0.41). There were no main effects of condition (*P* = 0.41) nor an interaction (*P* = 0.17) for *T*_c_, or *T*_sk_ (main effect condition: *P* = 0.44; interaction: *P* = 0.68); however, both Tc and Tsk significantly increased over time (*P* < 0.001, Fig. 2A & B).

### Interleukin-6 and receptor responses

**Fig. 3:**
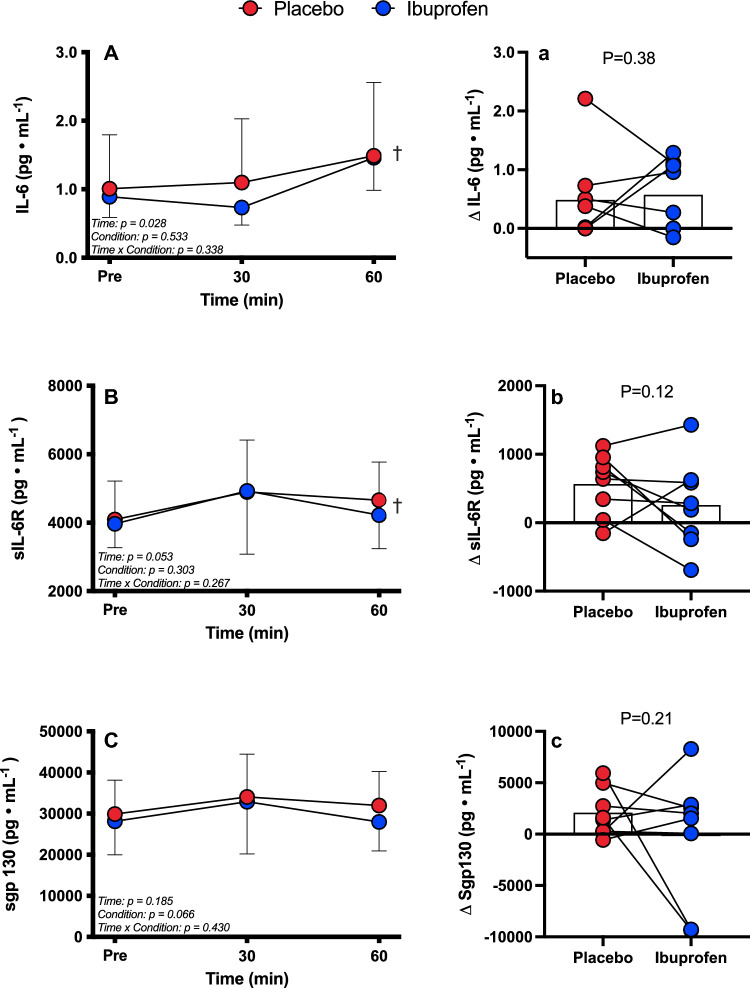
Release of IL-6 (**A**), delta (pre- to post-trial) IL-6 (**a**) sIL-6R (**B**), delta sIL-6R (**b**), sgp130 (**C**) and delta sgp130 **(c)**. *N* = 8, data presented as mean ± SD during 60 min cycling in the heat after ingestion of either placebo (red) or Ibuprofen (blue). *IL-6* Interleukin-6, *sIL-6R* soluble IL-6 receptor, *sgp130* soluble glycoprotein 130; ^†^main effect for time (*P* ≤ 0.05)

There was a main effect of time for IL-6 (*P* = 0.03, ES = 0.44, Fig. 3A) and sIL-6R (*P* = 0.05, ES = 0.43, Fig. 3B), but no main effect for condition or interactions for either (*P* ≥ 0.27). Interestingly, there was no main effect for time (*P* = 0.18) or condition (*P* = 0.07), nor interaction (*P* = 0.43) for sgp130 (Fig. 3C). At the end of exercise, the increase in IL-6 (Fig. 3A), sIL-6R (Fig. 3B) and sgp130 (Fig. 3C) was not different between conditions (*P* ≥ 0.05).

### Cortical activity and cerebral oxygenation

**Fig. 4:**
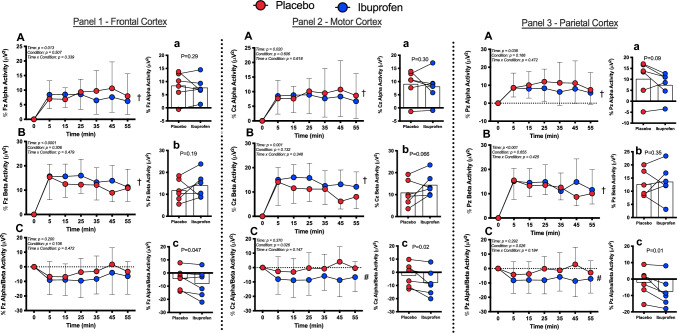
Percent change from baseline in alpha (**A**), average alpha throughout trial (**a**), beta (**B**), average beta throughout trial (**b**), alpha/beta ratio (**C**) and average alpha/beta ratio throughout trial (**c**) activity in the frontal cortex (Panel 1); motor cortex (Panel 2) and parietal cortex (Panel 3). *N* = 6 due to excessive artefact in two subjects. Data presented as mean ± SD during 60 min cycling in the heat after ingestion of either placebo (red) or Ibuprofen (blue). ^†^Main effect for time (*P* ≤ 0.04); ^#^main effect for trial (*P* ≤ 0.03)

**Fig. 5:**
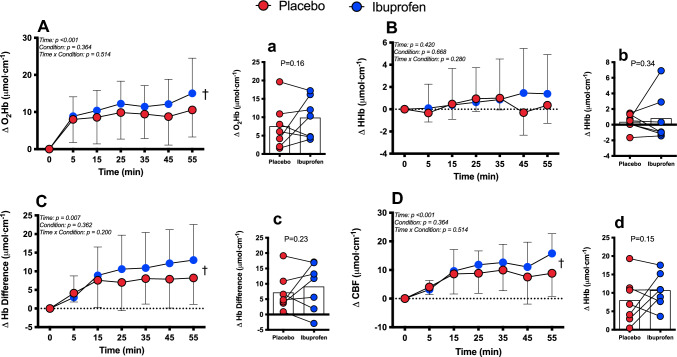
The absolute change in oxyhaemoglobin (O_2_Hb, **A**), average O_2_Hb (**a**), change in deoxyhaemoglobin (Hhb, **B**), average Hhb (**b**), change in haemoglobin difference (ΔHb_difference_, **C**), average Hb_difference_ (**c**), change in cerebral blood flow (ΔCBF, **D**), average CBF (**d**). *N* = 7 due to excessive artefact in one subject. Data presented as mean ± SD during 60 min cycling in the heat after ingestion of either placebo (red) or Ibuprofen (blue). ^†^Main effect for time (*P* ≤ 0.04)

The EEG activity for the *α* and *β* waves is shown in Fig. 4. The percent increase in *α* and *β* wave activity in the frontal, motor and parietal cortices (*n* = 6, due to excessive artefact in two subjects) was not different between conditions (*P* ≥ 0.16) but there was a main effect for time (*P* ≤ 0.04) revealing a significant increase from baseline. Average *α* and *β* wave activity was not different between conditions at any location (*P* ≥ 0.07, ES range: 0.14–0.67, all small effects except for a medium ES for average beta activity in the motor cortex (ES = 0.67) and alpha activity in the parietal cortex (ES = 0.46)). The percent change in *α*/*β* activity was reduced in both conditions and was significantly different in the motor and parietal cortices (Fig. 4C; *P* < 0.03). Average percent change in *α*/*β* activity revealed a greater reduction in Ibuprofen across all sites (Fig. 4C; *P* ≤ 0.047, ES range: 0.35–0.60, all small effects except for a medium ES for average percent change, In *α*/*β* in the motor (ES = 0.52) and parietal cortices (ES = 0.60)). There was a main effect for time for the change in O_2_Hb, the ΔHb_difference_ and the change in cerebral blood flow (Fig. 5A, C, D; *P* < 0.01), but no main effect for condition or interactions (*P* ≥ 0.05). The average ΔO_2_Hb, Hb_difference_, Hhb and cerebral blood flow did not differ between conditions (*P* ≥ 0.18, ES range 0.28–0.48, all small effects except a medium effect (ES = 0.48) for average cerebral blood flow).

## Discussion

This study tested the hypothesis that ingesting 800 mg Ibuprofen would improve exercise performance and attenuate fatiguing behaviour during a self-paced (clamped RPE) exercise protocol in the heat but attenuate the IL-6 and associated receptor response. A secondary hypothesis was that ingestion of Ibuprofen would result in increased *β* wave activity and cerebral oxygenation, and attenuate the increase in *α* wave activity compared to a placebo control. The data reveal that ingestion of 800 mg of Ibuprofen 1 h prior to exercise in a hot (35 °C, 60% RH) environment does not improve exercise performance, indicated by similar levels of power output in both conditions (Fig. 1). In regard to our second hypothesis, our findings show that neither *α* nor *β* wave activity was increased in the motor cortex after ingesting 800 mg Ibuprofen (Fig. 4). However, the *α*/*β* ratio was reduced in the Ibuprofen trial in all three cortices (Fig. 4). In addition, ingestion of Ibuprofen prior to exercise did not augment cerebral oxygenation compared to a placebo as originally hypothesised. Collectively, these data suggest that ingestion of Ibuprofen prior to exercise is unlikely to be of ergogenic benefit, but may transiently modulate cortical activity. The reason for the lack of ergogenic effect is not readily apparent. We can only speculate that the Ibuprofen dosage used in the present study did not reach a threshold value required to exert its influence centrally. That is, the combined effect of Ibuprofen and exercise training is thought to protect against overproduction of pro-inflammatory cytokines which could in turn alter the neuronal functions related to exercise-induced fatigue (Lima et al. 2015). This is a likely possibility given that we found no differences in our inflammatory profile between placebo and Ibuprofen groups.

### Ibuprofen does not improve performance, modulate core temperature or attenuate fatiguing behaviour in the heat

NSAIDs are often ingested prior to exercise to help mitigate pain and improve performance (Brewer et al. 2014; Holgado et al. 2018; Wijck et al. 2012). In the present study, power output was not significantly different between Ibuprofen and placebo trials, decreasing by 12.9% and 14.3% over time, respectively. In contrast, acetaminophen has been shown to improve time to exhaustion during exercise in the heat (Mauger et al. 2010). The mechanism for this improvement was suggested to be that acetaminophen modulated pain perception so that peripheral locomotor fatigue is restricted to a critical threshold which attenuates afferent feedback. In the present study, we did not observe an improvement in exercise performance nor an associated effect on cardiovascular or thermoregulatory responses. Specifically, HR (Fig. 1C), *T*_c_ (Fig. 2A) and *T*_sk_ (Fig. 2B) did not change when Ibuprofen was ingested compared to a placebo. There were also no significant differences in RPE between trials. Participants reported an RPE of 15–17 (hard to very hard) at each 5 min time point which indicates that subjects maintained a conscious effort despite increased HR and reductions in PO over time. The average HR was ~ 80 and 77% of maximum HR for placebo and Ibuprofen, respectively. Likewise the average PO was 42% and 43% of maximum for placebo and Ibuprofen, respectively. These findings strongly suggest that our protocol maintained the physiological responses relative to the perceived effort with participants adjusting their response accordingly, despite Ibuprofen not exerting an effect.

### Acute Ibuprofen ingestion does not appear to affect plasma IL-6, or its soluble receptor concentrations during self-paced aerobic exercise in the heat

During mixed-mode (resistance and aerobic) exercise training, acute ingestion of Ibuprofen has been shown to attenuate the release of IL-6 (Wherry et al. 2021). There was no effect of Ibuprofen on plasma concentrations of IL-6 or *T*_c_ response in the present study. Moreover, plasma concentrations of IL-6 in the present study were much lower in both conditions compared to previous reports (Starkie et al. 2005; Peake et al. 2008), despite the combination of high-intensity exercise and heat stress. Nevertheless, our data do show significant increases in IL-6 in both conditions, and a similar increase in *T*_c_. Thus, it can be concluded that there was no independent effect of Ibuprofen on IL-6 on *T*_c_ responses. This is a key finding as it may not be useful or prudent for an athlete, recreational or otherwise, to engage in the use of NSAID for ergogenic purposes within the exercise intensity and ambient conditions used in the present study.

To the best of our knowledge, sIL-6R and sgp130 responses to self-paced exercise during heat stress have had relatively little examination except in our previous study which examined the effects of different-intensity (RPE 12 and RPE 16) exercise and thermal stress (22 °C or 35 °C, 60% RH) on IL-6, and its soluble receptors using the same exercise protocol (Vargas and Marino 2018). Receptor responses in the prior study were similar between all trials. Interestingly, the sIL-6R response was slightly lower in the present study compared with the RPE 16 trials in the previous study. Nevertheless, the similar responses between both soluble receptors again suggests that Ibuprofen does not directly affect changes in performance specifically through IL-6 trans-signalling.

### Ibuprofen may exert central effects on neuronal activity

The present findings reveal that Ibuprofen did not specifically affect absolute *α* or *β* wave activity in the frontal, motor or parietal cortices, but did augment the change in *α*/*β* activity in the frontal, motor and parietal cortices (Fig. 4). Interestingly, the mechanisms contributing to the similar change in *α*/*β* activity at each cortice were different. Specifically, the difference in *α*/*β* ratio was due to slightly (though not significant) augmented *β* waves in the motor cortex, yet attenuated *α* waves in the frontal and parietal cortices. This is particularly interesting in the context of the present study because it may suggest that Ibuprofen preferentially maintains motor output (Bailey et al. 2008) whilst attenuating or inhibiting underlying neural processes (Hilty et al. 2010; Robertson and Marino 2016). That this did not translate to greater power output in the Ibuprofen trial, however, is difficult to reconcile, but may be due to the exercise protocol we employed. That is, although we clamped the protocol at a pre-determined RPE, it is possible that underlying neural processes that contribute to the perception of RPE represent some of the alpha waves that are measured in the EEG, but likely represent other processes as well. Hence, although there appears to be an attenuation of these neural processes (which could be interpreted as having the potential to reduce RPE), it did not translate to greater power output; however, this is speculative. Prior studies have used incremental exercise tests to exhaustion, maximal hand grip or time trial methodologies. Thus, understanding the relation between EEG signals representing motor output and those representing underling neural processes contributing to RPE during an exercise protocol that is based on overall perceived exertion requires further investigations. However, we cannot discount that neuronal activity in the motor cortex might be preferentially maintained as shown even at high exercise intensities or at exhaustion (Robertson and Marino 2015). In contrast to the EEG findings, there were no significant findings related to cerebral oxygenation, deoxygenation or haemoglobin differences when Ibuprofen was ingested (Fig. 5). These results overall suggest that Ibuprofen may exert a central effect on neuronal activity, at least as observed in EEG data, but this did not translate to differences in cerebral blood flow.

## Limitations

There are several considerations that should be noted with respect to our findings. First is that this study was underpowered with observed power for the present data ranging from 0.12 to 0.86. Although our findings should be cautiously interpreted, further research is warranted as others have highlighted that analgesic response might be dependent on the differences in intensity and exercise protocols utilised to evaluate its effects (Garcin et al. 2005). In addition, our intra-assay CV for IL-6 is borderline acceptable (acceptable for HCYTOMAG-60 K = within 2–13%) and sgp130 is slightly above acceptable range (acceptable for HSCRMAG-32 K ≤ 10%), suggesting there may have been a small issue with the pipetting of samples during analysis. These data should also be interpreted with caution and further research is recommended.

We only included males in our sample due to perceived difficulties testing women within a very tight testing schedule. Although time is not a particularly justifiable reason to not include women, we recognise this as a prominent limitation and are supportive of equal gender representation in future studies. It is also possible that the clamped RPE protocol may not have been sensitive enough to identify whether or not performance and fatiguing behaviour would be affected by the ingestion of Ibuprofen prior to exercise. Rather, it may be possible to employ a protocol that can improve experimental control, such as a time trial or time to exhaustion protocol. However, a clamped RPE protocol is an ecologically appropriate protocol for many training and performance contexts; thus, our findings are still informative for the practise of ingesting NSAIDs prior to exercise.

An additional consideration is that we used a dose of Ibuprofen equivalent to the maximal concentration that is suggested on an over-the-counter Ibuprofen package (i.e. 800 mg). This method was chosen for both the safety of the subjects, and for the ecological validity in settings where athletes are likely to follow the packaging dosage recommendations. Our mean dose was 9.4 ± 0.7 mg/kg (range 8.1–10.3). Nevertheless, standardising the dosage across all subjects may result in different outcomes when Ibuprofen is ingested prior to exercise and could be considered for future research.

## Conclusion

In conclusion, ingestion of an over-the-counter dose of Ibuprofen did not alter the plasma concentration of IL-6, sIL-6R or sgp-130 during exercise in the heat. Ibuprofen also did not improve PO, but total *α*/*β* activity across the frontal, motor and parietal cortices was reduced to a greater extent during the protocol when Ibuprofen was ingested. Based on these findings, Ibuprofen does not appear to modulate trans-signalling of afferent sensory feedback during self-paced exercise in the heat, despite altering neuronal activity.

## Data Availability

The data that support the findings of this study are available from the corresponding author.

## References

[CR1] Babiloni C et al (2009) Ibuprofen treatment modifies cortical sources of EEG rhythms in mild Alzheimer’s disease. Clin Neurophysiol 120:709–718 19324592 10.1016/j.clinph.2009.02.005

[CR2] Bailey SP, Hall EE, Folger SE, Miller PC (2008) Changes in EEG during graded exercise on a recumbent cycle ergometer. J Sports Sci Med 7:505–511 24149958 PMC3761919

[CR3] Billaut F, Davis JM, Smith KJ, Marino FE, Noakes TD (2010) Cerebral oxygenation decreases but does not impair performance during self-paced, strenuous exercise. Acta Physiol 198:477–486 10.1111/j.1748-1716.2009.02058.x 19912150

[CR4] Borg GA (1982) Psychophysical bases of perceived exertion. Med Sci Sports Exerc 14:377–381 7154893 10.1249/00005768-198205000-00012

[CR5] Brewer CB, Bentley JP, Hallam JS, Woodyard CD, Waddell DE (2014) Use of analgesics for exercise-associated pain. J Strength Cond Res 28:74–81 23542880 10.1519/JSC.0b013e318291ba98

[CR6] Bushra R, Aslam N (2010) An overview of clinical pharmacology of ibuprofen. Oman Méd J 25:155–161 22043330 10.5001/omj.2010.49 PMC3191627

[CR7] Ftaiti F, Kacem A, Jaidane N, Tabka Z, Dogui M (2010) Changes in EEG activity before and after exhaustive exercise in sedentary women in neutral and hot environments. Appl Ergon 41:806–811 20206916 10.1016/j.apergo.2010.01.008

[CR8] Garcin M, Mille-Hamard L, Billat V, Humbert L, Lhermitte M (2005) Influence of acetaminophen consumption on perceived exertion at the lactate concentration threshold. Percept Mot Skills 101:675–683 16491671 10.2466/pms.101.3.675-683

[CR9] Gorski T et al (2011) Use of NSAIDs in triathletes: prevalence, level of awareness and reasons for use. Br J Sports Med 45:85 19666628 10.1136/bjsm.2009.062166

[CR10] Gray SR et al (2009) The response of circulating levels of the interleukin-6/interleukin-6 receptor complex to exercise in young men. Cytokine 47:98–102 19527938 10.1016/j.cyto.2009.05.011

[CR11] Harden LM et al (2014) Critical role for peripherally-derived interleukin-10 in mediating the thermoregulatory manifestations of fever and hypothermia in severe forms of lipopolysaccharide-induced inflammation. Pflügers Arch - Eur J Physiol 466:1451–1466 24114176 10.1007/s00424-013-1371-4

[CR12] Hilty L, Jäncke L, Luechinger R, Boutellier U, Lutz K (2010) Limitation of physical performance in a muscle fatiguing handgrip exercise is mediated by thalamo-insular activity. Hum Brain Mapp 32:2151–2160 21154789 10.1002/hbm.21177 PMC6870353

[CR13] Holgado D, Hopker J, Sanabria D, Zabala M (2018) Analgesics and Sport Performance: Beyond the Pain-Modulating Effects. PM&R 10:72–82 28782695 10.1016/j.pmrj.2017.07.068

[CR14] Jones SA, Rose-John S (2002) The role of soluble receptors in cytokine biology: the agonistic properties of the sIL-6R/IL-6 complex. Biochimica Et Biophysica Acta Bba - Mol Cell Res 1592:251–263 10.1016/S0167-4889(02)00319-1 12421670

[CR15] Lim CL, Mackinnon LT (2006) The roles of exercise-induced immune system disturbances in the pathology of heat stroke. Sports Med 36:39–64 16445310 10.2165/00007256-200636010-00004

[CR16] Lima FD et al (2015) Ibuprofen intake increases exercise time to exhaustion: a possible role for preventing exercise-induced fatigue. Scand J Med Sci Sports 26:1160–1170 26589249 10.1111/sms.12549

[CR17] Mauger AR, Jones AM, Williams CA (2010) Influence of acetaminophen on performance during time trial cycling. J Appl Physiol 108:98–104 19910336 10.1152/japplphysiol.00761.2009

[CR18] Nielsen B, Hyldig T, Bidstrup F, González-Alonso J, Christoffersen GRJ (2001) Brain activity and fatigue during prolonged exercise in the heat. Pflugers Arch 442:41–48 11374067 10.1007/s004240100515

[CR19] Nieman DC et al (2005) Muscle damage is linked to cytokine changes following a 160-km race. Med Sci Sports Exerc 37:S336 10.1016/j.bbi.2005.03.008 16061149

[CR20] Ogoh S, Ainslie PN (2009) Cerebral blood flow during exercise: mechanisms of regulation. J Appl Physiol 107:1370–1380 19729591 10.1152/japplphysiol.00573.2009

[CR21] Pauw KD et al (2013) Guidelines to classify subject groups in sport-science research. Int J Sports Physiol Perform 8:111–122 23428482 10.1123/ijspp.8.2.111

[CR22] Peake J et al (2008) Body temperature and its effect on leukocyte mobilization, cytokines and markers of neutrophil activation during and after exercise. Eur J Appl Physiol 102:391–401 17962974 10.1007/s00421-007-0598-1

[CR23] Perrey S (2008) Non-invasive NIR spectroscopy of human brain function during exercise. Methods 45:289–299 18539160 10.1016/j.ymeth.2008.04.005

[CR24] Ranamathan NL (1976) A new weighting system for mean surface temperature of the human body. J Appl Physiol 41:256–258 14173555 10.1152/jappl.1964.19.3.531

[CR25] Rasmussen P et al (2010) Reduced muscle activation during exercise related to brain oxygenation and metabolism in humans. J Physiol 588:1985–1995 20403976 10.1113/jphysiol.2009.186767 PMC2901984

[CR26] Rhind SG et al (2009) Cytokine induction during exertional hyperthermia is abolished by core temperature clamping: neuroendocrine regulatory mechanisms. Int J Hyperth 20:503–516 10.1080/02656730410001670651 15277023

[CR27] Robertson CV, Marino FE (2015) Prefrontal and motor cortex EEG responses and their relationship to ventilatory thresholds during exhaustive incremental exercise. Eur J Appl Physiol 115:1–10 25917836 10.1007/s00421-015-3177-x

[CR28] Robertson CV, Marino FE (2016) A role for the prefrontal cortex in exercise tolerance and termination. J Appl Physiol 120:464–466 26404617 10.1152/japplphysiol.00363.2015

[CR29] Rose-John S (2012) IL-6 trans-signaling via the soluble IL-6 receptor: importance for the pro-inflammatory activities of IL-6. Int J Biol Sci 8:1237–1247 23136552 10.7150/ijbs.4989 PMC3491447

[CR30] Schlader ZJ, Stannard SR, Mündel T (2011a) Evidence for thermoregulatory behavior during self-paced exercise in the heat. J Therm Biol 36:390–396 10.1016/j.jtherbio.2011.07.002

[CR31] Schlader ZJ, Stannard SR, Mundel T (2011b) Exercise and heat stress: performance, fatigue and exhaustion—a hot topic. Br J Sports Med 45:3–5 19846428 10.1136/bjsm.2009.063024

[CR34] Starkie RL, Hargreaves M, Rolland J, Febbraio MA (2005) Heat stress, cytokines, and the immune response to exercise. Brain Behav Immun 19:404–412 16061150 10.1016/j.bbi.2005.03.005

[CR35] Tucker R (2009) The anticipatory regulation of performance: the physiological basis for pacing strategies and the development of a perception-based model for exercise performance. Br J Sports Med 43:392–400 19224911 10.1136/bjsm.2008.050799

[CR36] van Wijck K et al (2012) Aggravation of exercise-induced intestinal injury by Ibuprofen in Athletes. Med Sci Sports Exerc 44:2257–2262 22776871 10.1249/MSS.0b013e318265dd3d

[CR37] Vargas N, Marino F (2016) Heat stress, gastrointestinal permeability and interleukin-6 signaling—implications for exercise performance and fatigue. Temperature 3:240–251 10.1080/23328940.2016.1179380 PMC4964994 27857954

[CR38] Vargas N, Marino F (2018) Neuroinflammation, cortical activity, and fatiguing behaviour during self-paced exercise. Pflügers Archiv – Eur J Physiol 470:413–426 29159538 10.1007/s00424-017-2086-8

[CR39] Wallenstein MC (1985) Differential effects of prostaglandin synthetase inhibitors on EEG in rat. Eur J Pharmacol 111:201–209 3926513 10.1016/0014-2999(85)90757-5

[CR40] Wherry SJ, Wolfe P, Schwartz RS, Kohrt WM, Jankowski CM (2021) Ibuprofen taken before exercise blunts the IL-6 response in older adults but does not alter bone alkaline phosphatase or c-telopeptide. Eur J Appl Physiol 121:2187–2192 33876259 10.1007/s00421-021-04691-8 PMC11416062

